# Understanding the acceptability, barriers and facilitators for chlamydia and gonorrhoea screening in technical colleges: qualitative process evaluation of the “Test n Treat” trial

**DOI:** 10.1186/s12889-020-09285-1

**Published:** 2020-08-08

**Authors:** Charlotte Fleming, Vari M. Drennan, Sarah Kerry-Barnard, Fiona Reid, Elisabeth J. Adams, S. Tariq Sadiq, Rachel Phillips, Wendy Majewska, Emma M. Harding-Esch, Emma C. Cousins, Freya Yoward, Pippa Oakeshott

**Affiliations:** 1 Population Health Research Institute, St George’s, University of London, London, UK; 2 Centre for Health & Social Care Research, Kingston University & St George’s, University of London, London, UK; 3 School of Population Health and Environmental Sciences, King’s College London, London, UK; 4 Aquarius Population Health Limited, London, UK; 5 Institute for Infection and Immunity, St George’s, University of London, London, UK; 6 WEM Consultancy Ltd., London, UK; 7 Public Health England, London, UK

**Keywords:** Adolescent, Students, Screening, Technical colleges, Chlamydia, Sexually transmitted diseases, Sexual behaviour, Qualitative

## Abstract

**Background:**

Low uptake of sexually transmitted infection testing by sexually active young people is a worldwide public health problem. Screening in non-medical settings has been suggested as a method to improve uptake. The “Test n Treat” feasibility trial offered free, on-site rapid chlamydia/gonorrhoea tests with same day treatment for chlamydia (and gonorrhoea treatment at a local clinic,) to sexually active students (median age 17 years) at six technical colleges in London. Despite high rates of chlamydia (6% prevalence), uptake of testing was low (< 15%). In a qualitative study we explored the acceptability, including barriers and facilitators to uptake, of on-site chlamydia screening.

**Methods:**

In 2016–17 we conducted a qualitative study in the interpretative tradition using face to face or telephone semi-structured interviews with students (*n* = 26), teaching staff (*n* = 3) and field researchers (*n* = 4). Interviews were digitally recorded, transcribed and thematically analysed.

**Results:**

From the student perspective, feelings of embarrassment and the potential for stigma were deterrents to sexually transmitted infection testing. While the non-medical setting was viewed as mitigating against stigma, for some students volunteering to be screened exposed them to detrimental judgements by their peers. A small financial incentive to be screened was regarded as legitimising volunteering in a non-discrediting way. Staff and researchers confirmed these views. The very low level of knowledge about sexually transmitted infections influenced students to not view themselves as candidates for testing. There were also suggestions that some teenagers considered themselves invulnerable to sexually transmitted infections despite engaging in risky sexual behaviours. Students and researchers reported the strong influence peers had on uptake, or not, of sexually transmitted infection testing.

**Conclusions:**

This study offers new insights into the acceptability of college-based sexually transmitted infection screening to young, multi-ethnic students. Future studies in similar high risk, hard to reach groups should consider linking testing with education about sexually transmitted infections, offering non stigmatising incentives and engaging peer influencers.

## Background

*Chlamydia trachomatis* (CT, chlamydia) is the most commonly reported bacterial sexually transmitted infection (STI) in the United Kingdom (UK) [1, 2]. Estimated population-based prevalence rates in those aged 16–24 years are 3.1% for women and 2.3% for men [2] with higher rates in ethnically diverse, sexually active teenagers and in those from less affluent backgrounds [1]. Often asymptomatic, infection with chlamydia can lead to pelvic inflammatory disease, epididymitis and infertility. It is treated with a course of antibiotics. *Neisseria gonorrhoeae* (GC, gonorrhoea) is a STI,which causes similar reproductive sexual health long term problems, is much less common (< 0.1% prevalence in women and men) [1] but with increasing rates in the London region of the UK [3] and with growing concerns regarding antimicrobial resistance [4]. Gonorrhoea is harder to treat and usually managed by specialists. England has a free national screening programme for chlamydia, which is offered opportunistically through primary care and sexual health services to all sexually active people aged 15 to 24 years at least annually and on change of partner [1]. However, uptake of chlamydia testing is suboptimal and declining [1]. Most young people diagnosed with chlamydia in England in the NATSAL-3 survey had not accessed sexual health services in the past year [2]. Multiple factors such as embarrassment and the stigma associated with STIs as well as problems with access are known to inhibit uptake of testing [1, 5–7]. Identifying ways of increasing uptake of STI testing by different populations remains a public health priority. Provision of testing in non-medical settings has been identified as one approach [8, 9].

Technical colleges (known as further education colleges in the UK) are a non-medical setting in which there are large numbers of a high-risk population – sexually active teenagers. These colleges offer both academic and practical courses for people aged ≥16 years and take many students from socio-economically deprived backgrounds. Technical colleges do not usually have on-site health centres. Most research on student populations in high income countries has focused on high schools and higher education institutions (universities). Studies include STI screening programmes delivered by school health staff [10, 11] or surveys of sexual behaviour, knowledge, attitudes and uptake of STI testing [12, 13]. However, there is very limited research with technical college students. One survey in the Netherlands, where STI testing is free and easily available in health care settings, suggested that low uptake of testing was most closely associated with attitudes, including perceptions of difficulties with access [14]. There is an urgent need for research on whether bringing novel rapid CT and GC tests to this high risk population could improve detection and treatment rates [15].

The “Test n Treat” (TnT) trial investigated the feasibility of offering rapid 90-min on-site CT/GC testing, (with same day on-site treatment for chlamydia and referral to sexual health services for gonorrhoea), to students attending six technical colleges in London, UK in the academic year 2016–17 [16, 17]. The trial reported a high rate of chlamydia (6%) and gonorrhoea (1%) for that age group in a community setting [17]. However, the uptake of the on-site TnT testing was only 13% at one month and 10% at four months after recruitment [17]. This low uptake of the STI testing occurred despite the TnT feasibility trial incorporating evidence from young female, technical college student preferences regarding on-site STI screening [18], a successful pilot study [19], and involving students in the trial design [16, 17].

We conducted a qualitative process evaluation alongside the TnT trial. This was guided by the Medical Research Council’s framework for developing and testing complex interventions in order to evaluate the trial implementation, to offer explanatory theories as to the success or failure, and to inform future research and/or service provision decisions [20]. We explored the following research questions:

- Is the provision of rapid chlamydia and gonorrhoea testing in technical colleges viewed as acceptable and appropriate by students?
- What are the barriers and facilitators to uptake as perceived by young people, teaching staff and on-site researchers?
- What factors or strategies might improve uptake of rapid chlamydia and gonorrhoea testing in technical colleges from the perspectives of young people, teaching staff and on-site researchers?

The inquiry was initially informed by two sets of theories. The first was Goffman’s theory of stigma [21] in which socially discreditable characteristics mark an individual as negatively different to others. The second theory was the construct of ‘candidacy’ [22]. Candidacy is used to describe an individual’s recognition of their eligibility for a service. It is argued that candidacy is an iterative process and subject to social influences and contexts [22].

## Methods

This study was undertaken in the interpretive research tradition, underpinned by the pragmatist paradigm which recognises multiple perceptions of social reality occurring within specific socio-cultural and historical contexts but is focused on problems and solutions [23]. Semi-structured interviews were conducted with students, college staff and researchers. The interviews were conducted with topic guides, drawn from the research questions and tailored to the participant groups. This also allowed for flexibility in capturing participant views [24] (Additional file 1).

### Participants

**Fig. 1:**
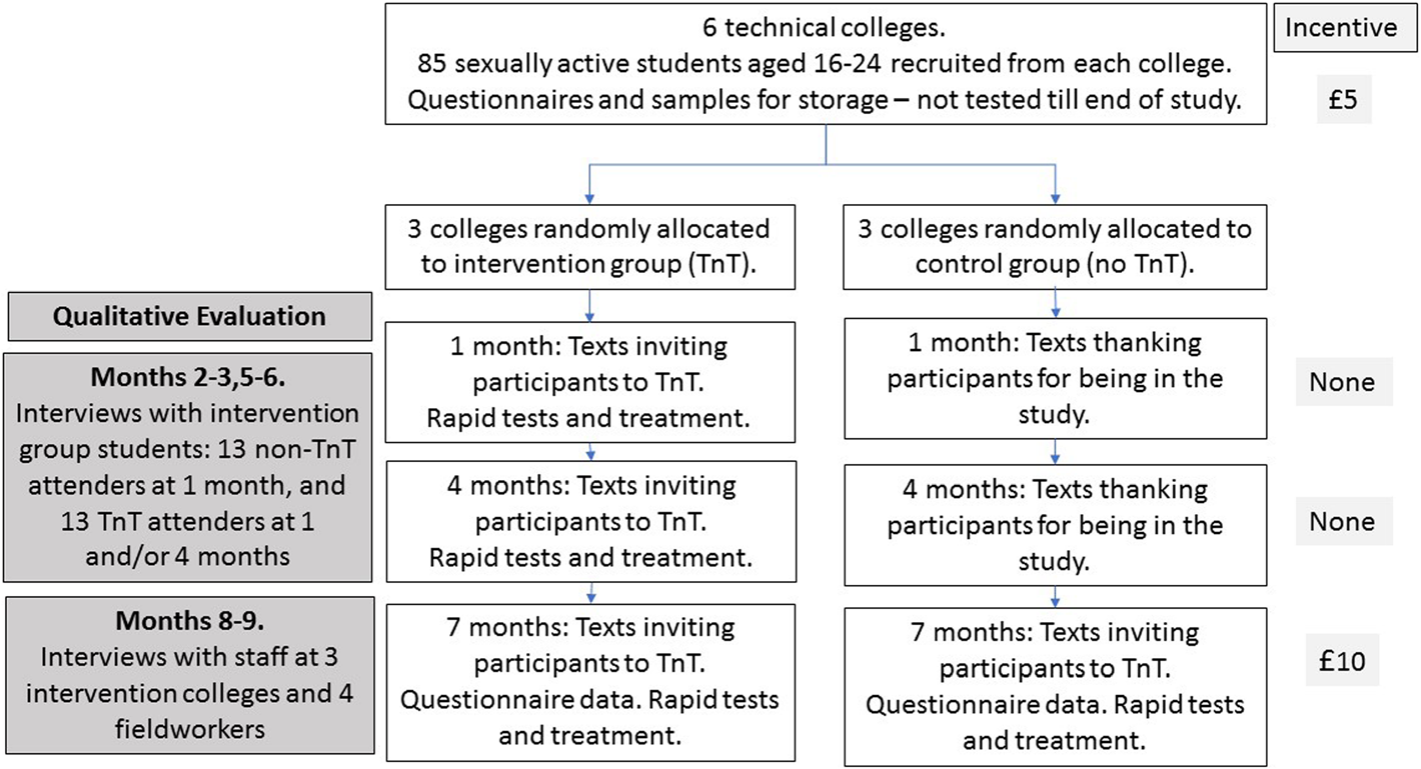
Design of the qualitative evaluation nested within the Test n Treat (TnT) feasibility trial

For the main TnT trial, 509 sexually active students aged 16–24 years were recruited from public areas at six colleges [17] (Fig. 1). Three colleges were then randomly assigned to receive the intervention, and the process evaluation was undertaken with participants in these colleges. All participants were aged over 16 years.

At baseline, participants completed questionnaires (including self-declared ethnicity), and provided genitourinary samples for storage (urines for males, self-taken vaginal swabs for females). They received £5 (roughly equivalent to 5 Euros/ 6 US dollars) as an honorarium at recruitment, and £10 at the seven months follow up test. There was no financial incentive for attending for TnT testing, as in the UK people are not usually paid for being tested for STIs.

Participating students in the three intervention colleges were texted invitations to on-site STI testing (TnT) one and four months after recruitment. Testing activities were undertaken in private rooms. Participants at all six colleges were texted invitations to attend the seven months follow up.

For the qualitative process evaluation in the three intervention colleges, a purposive sample [24] of male and female students across the age range of 16–24 years and ethnic backgrounds, were approached by text/telephone to volunteer to be interviewed after month one and four (Fig. 1). The sample included both those who had and also had not attended for screening. Participants were recruited until no new themes were identified. CF, a female health researcher, undertook these interviews between December 2016 and March 2017. In addition, CF interviewed the main TnT contact member of staff from these three colleges. These were staff who were supportive of the idea of providing such services within the college setting. VMD, a female health researcher, interviewed the four researchers who did the fieldwork (SKB, CF, EC and WM) and used reflective techniques in the interview for checking understanding and interpretation [24].

Interviews took place either in a private room at college or over the phone at a time that was convenient for the participant. Written informed consent for interviews was obtained at recruitment, and then oral consent was provided at the time of interview. Interviews lasted between 5 and 28 min, were digitally recorded with permission and backed with field notes. Recordings were transcribed, anonymised and then destroyed.

### Data analysis

Transcripts were read, re-read, coded and analysed using thematic analysis [25]. The analysis was informed by the study topic guide and the initial theoretical framing of the study. Data were coded line by line and then clustered manually to identify categories based on issues and themes. Data were then grouped in main analytic themes. Where data did not fit into existing themes, new ones were developed or existing ones modified until all data were grouped by theme by CF and VMD, resolving differences through discussion [25]. The analysis was further refined in discussion with the wider research team. Trustworthiness and credibility of the analysis were explored in meetings that included student representatives, and no further themes were identified.

Reporting conforms to Standards for Reporting Qualitative Research [26] and the checklist is provided in additional file 2.

## Results

We first describe the characteristics of the student participants and then report the themes within each of the research questions.

### The student participants

Interviews were carried out with 26 students: 13 TnT non-attenders and 13 TnT attenders for testing. Two further students who had agreed to be interviewed did not attend or respond to subsequent texts and calls. The mean age of respondents was 17.5 years, 62% were female, 50% described their ethnic group as black. Most participants (92%, 22/24) who responded to the question said they were heterosexual (Table 1).

**Table 1:** Demographic characteristics of 26 interviewed student participants

		TnT non-attenders *n* = 13	TnT attenders *n* = 13	Total *n* = 26
Characteristic		n (%)	n (%)	n (%)
Sex				
Male		4 (31)	6 (46)	10 (38)
Female		9 (69)	7(54)	16 (62)
Ethnicity				
White		4 (31)	3 (23)	7 (27)
Black		6 (46)	7 (54)	13 (50)
Asian		2 (15)	1(8)	3 (12)
Other		1 (8)	2(15)	3 (12)
Age (years)				
16–18		9 (70)	12 (92)	21 (81)
19–21		2 (15)	1 (8)	3 (12)
22–24		2 (15)	0 (0)	2 (8)
Mean (SD) age (years)		17.8 (2.3)	17.1 (1.1)	17.5 (1.8)
Sexual Partners				
Females	Sex with men only	7 (53)	7 (53)	14 (54)
	Sex with women only	1(8)	0	1 (4)
	Sex with both sexes	0	0	0
	Prefer not to say	1(8)	0	1 (4)
Males	Sex with women only	2(8)	6(23)	8(31)
	Sex with men only	1(8)	0	1(4)
	Sex with both sexes	0	0	0
	Prefer not to say	1(8)	0	1 (4)

### Student perceptions of the acceptability or otherwise of on-site STI testing

The TnT study itself was viewed very positively by most of the students interviewed (*n* = 25/26). Attenders thought the service was ‘*amazing*’ (interviewee 220), ‘*educational*’ (interviewee 117), ‘*friendly*’ (interviewee 429), and ‘*helpful*’ (interviewee 131). Even those who did not attend considered it was a valuable service within a non-medical setting.

“*I think the service that you provide is actually very good because, like, most kids ,I think, they would be too shy to, like, go out and get checked to see if they have any like STD’s or anything. So I think, like, you’re supporting them a lot by coming here and asking them if they need to because then it’s not like they have to go out of their way to do it.”* Interviewee 451 (male, age group 16–18 years,TnT non-attender).

Reasons reported by participants for not attending for testing at TnT visits were similar for both males and females; they did not attend because they were not in college that day or were busy in class or working on college assignments. Others said they did not get the text message or forgot. Despite the verbal and written explanations given by the research team, many participants did not understand that the first samples they provided at recruitment were stored and not tested for STIs. This was one reason for subsequent non-attendance.

“*I think it’s because once you’ve done it* [provided a sample] *once, people don’t really want to do it again and again. It seems, it’s like a drag*.” Interviewee 069 (female, age group 16–18 years, TnT non-attender).

The students also described the types of factors, outside of the TnT study design, that made on-site STI testing less acceptable and were barriers to take up. These are reported in the next section.

### Perceived barriers to uptake of the on-site STI testing

The themes identified as barriers included: feelings of embarrassment and perceived stigma, the influence of peers, lack of knowledge of STIs, perceptions of invulnerability and the potential of surveillance.

#### Embarrassment and perceived stigma

Feelings of embarrassment were perceived by students as a significant barrier for themselves and other students to taking the STI test.

“*I think it’s the embarrassing side of it, like, people just, I don’t know, like people just sort of are embarrassed about these things*.” Interviewee 429 (female, age group 16–18 years, TnT attender).

College staff also considered embarrassment was an issue amongst students. For some students, volunteering for STI testing was reported to be more than embarrassing, it was considered potentially shameful. The risk of being observed by people who knew them at either sexual health clinics, or within college for the TnT study, was described as a deterrent. Students observed that the act of volunteering for the test brought the risk of being thought to have an STI by peers. They described this as likely to be viewed negatively by peers and would be discrediting to an individual. There was some indication that there were two types of discredit, the first was that an STI made the individual unclean and not someone to interact with:

*“Don’t want to be looked on as the dirty person*.” Interviewee 104 (male, age group 16–18 years, TnT non-attender).

The second type of discredit concerned negative judgements about the individual’s sexual behaviour:

“*Some people can’t handle other people talking about them, so they feel like if they go and get done* [tested]*, people will talk and judge him or her*.” Interviewee 240 (female, age group 16–18 years, TnT attender).

The researchers were aware of the potential embarrassment and desire for confidentiality by the students. They described the ongoing tension between publicising that TnT was available and keeping the testing confidential.

*“So we* (the researchers) *discussed a number of times; ‘should we be in a discrete or public location? Would a discrete place be better?’ It felt right not to expose students to potential stigma, but it needed to be public enough to make sure students found us. So we ended up trying to do both”.* Researcher C.

In part this tension was created through the public nature of the recruitment, but there were also challenges in that private rooms were not always available within the college for undertaking the testing. Educational uses of rooms were always prioritised. The researchers gave examples about how the engagement with students kept them alert to feelings of embarrassment and potential stigma. For example, students were reported to not want the word “*chlamydia*” to appear in their TnT invitation texts in case someone else read them. The extent to which communications to students via mobile phones were secure and confidential was reported as an ongoing issue. The researchers described phone numbers being inaccurate, ceasing to work and, in some instances, students without functioning mobile phones gave other people’s (e.g. mother’s/aunt’s) phone numbers.

#### The influence of peers

Students, college staff and researchers all described how the views of peers were important and could act as barriers to taking the test. Recruitment and engagement with students mostly took place at break periods. The researchers described how there would be large groups of loud, boisterous teenagers who would approach the study team and this took some managing. Students rarely attended alone. Researchers observed that often one student in the group would be influential with negative comments about sexual activity or diseases which would then dissuade the whole group from participating.

*“It was often difficult particularly with large groups of boys, you know ‘showboating’ around, making negative comments and jokes about sexual diseases. I’m pretty sure this made it difficult for other students to approach the TnT table*.” Researcher D,

#### Lack of knowledge of STIs

Limited knowledge of STIs was considered by student participants to be an important barrier for themselves and their peers to taking up the offer of STI testing. Many participants were very open in declaring their limited understanding of STIs and their potential long-term consequences. Only 15% (4/26) knew that chlamydia could cause infertility.

“*I don’t know anything* [about STIs] *to be honest with you*.” Interviewee 220 (male, age range 16–18 years, TnT attender).

Some attributed their (and their peers) limited understanding of STIs to the lack of formal education on the subject. All participants said they had received sexual health education in school but this did not usually include STIs.

“*It was just mostly about putting on condoms … .. not to get pregnant, that’s mostly what they* [teachers in school] *talked about*.” Interviewee 217 (male, age group 16–18 years, TnT attender).

A lack of knowledge about asymptomatic STIs was also evident in those who reported that their peers only went for testing on noticing symptoms.

“*I think they wait for symptoms before they get tested*.” Interviewee 104 (male, age group 16–18 years, TNT non-attender).

Like the students, the college staff considered that sexual health education in schools was “*hit and miss*” with little attention to STIs. One suggestion was that colleges could teach about STIs in compulsory personal and sexual health education lessons which some students aged 16–19 years needed to attend in order to claim their government funded bursary.

#### The potential for surveillance

Amongst the barriers that the college staff identified were some students’ concerns that testing was a form of surveillance on their behaviour. They recounted student fears that information about participating would be relayed to their parents, or their urine might be tested for drugs.

“*There were some doubts from students about what would happen to their urine sample, whether it might be tested for other things and whether the ‘conspiracy police’ would tell their parents what they might have been doing*.” Lecturer A.

Some students echoed this concern about disclosure to parents, recounting anxiety in being asked for their home address in case anything about TnT or their results was sent to the home where a parent might read it.

#### Perceptions of invulnerability to STIs

Some students offered the view that their peers considered themselves as somehow invulnerable to getting infected, and this was a barrier as they did not consider themselves as candidates for testing:

“*I know people that like, they don’t, when they have sex they don’t have protected sex, they feel like ‘I’m clean. I’ve slept with so many people, I’ve never had STD’s’. Duh. They don’t know about diseases, how to prevent it*.” Interviewee 255 (female, age group 16–18 years, TnT attender).

However, some of the student participants described themselves and peers as aware of their need to be tested which we now discuss within the other themes reported to facilitate testing:

### Perceived facilitators for uptake of on-site STI testing

Themes identified as facilitators to uptake of the testing were: knowledge of the personal risk of STIs, the influence of peers, the non-medical setting and incentives.

#### Knowledge of the personal risk of STIs

While many student participants reported lack of knowledge of STIs as a barrier, some indicated they had enough knowledge to perceive themselves as at risk of STIs. Many of those who attended wanted a check-up as they had been with a new partner or multiple partners:

*“I was being with boys* [having sex] *a lot, you know, and I saw you* [recruiting for TnT] *and thought I needed a test, yes*.” Interviewee 429 (female, age group 16–18 years, TnT attender).

Some students reported that they persuaded friends to take up the TnT offer of testing as they knew they were potential candidates because they had had recent unprotected sex or sex with a new partner.

#### The influence of peers in facilitating STI testing

While the influence and views of peers could be a barrier to testing as described above, conversely, peers were also reported as important in encouraging take up of the TnT test. Many TnT attendee participants described their attendance as part of a group of friends:

“*One of my mates said you might as well do it. I was like, OK, I might as well see as well. So that’s why I did it*.” Interviewee 131 (male, age group 16–18 years, TnT attender).

The researchers also observed that certain individuals were influential in up-take of the test by groups of friends. They also noticed that some staff were effective in advocating uptake of the testing. In one college a male security guard was active in encouraging male students to use the TnT testing services.

#### The non-medical setting for STI testing as a facilitator

Some student participants perceived the TnT trial within the colleges as mitigating against the negative perceptions and connotations of STI testing. Students considered that having access to STI testing at college was positive and viewed it as more confidential and less likely to attract discrediting judgements by others:

“*They would use it more than the sexual health clinic because you know the clinic is labelled as they know that you’ve got STD’s. If you go into like a room -it’s college - you’re going into college*.” Interviewee 194 (female, age group 16–18 years, TnT non-attender).

#### The role of incentives as facilitating STI testing

Students, college staff and researchers all considered that the small value financial honorarium was a key facilitator to testing. The college staff suggested that students had many competing priorities and incentives were required:

“*It’s difficult to get students to do things … without an incentive. The students are busy and they are hard to pin down and their mobile* [telephone] *number has changed. The draw of hanging out with their friends is always going to be more attractive …*. T*hey are doing us a favour is probably how it seems to them*.” Lecturer C.

Finance (or rather their limited access to it) was important to many of the students. Researchers reported that £5 was a very tangible incentive; although of small value it was a ‘*big deal*’ to the students. Even though the money was not mentioned in the study recruitment poster, as the recruitment day went on students arrived at the TnT table expecting £5. Researchers viewed the money as a strong incentive, also giving some students ‘*license*’ to attend. However, the money also created some problems in that some students tried to ‘*game*’ the system returning more than once to participate and pretending they were someone else.

Some students also considered that the monetary incentive gave a legitimate reason to be tested. Money was considered to mitigate against the social stigma attached to STI testing but importantly it was viewed as an incentive not associated with sexual activity. By contrast, some young women described incentives associated with sex as potentially discrediting:

“*Because if it’s money people won’t really mind. But if it’s condoms, then they are going to be, like, ‘Who are you going to use it for?’*.” Interviewee 445 (female, age group 16–18 years, TnT non-attender).

However, some female students considered free condoms would be attractive incentives to male students. A college staff member commented that when there had been some on-site opportunities to obtain free condoms (no longer available at the time of the research) many male students had taken up the service.

### Views on future strategies to increase the uptake of STI testing in research like TnT

Three main themes emerged on this question: educational accompaniment to the offer of STI testing, publicity and practicalities, and the use of incentives.

#### Education to accompany testing

Some students, the college staff and the researchers considered that a simultaneous or just prior, educational initiative on sexual health, including STIs, would increase the knowledge level in students. This in turn, they speculated, would help students recognise that they were at risk and encourage them to take up the offer of testing. Suggestions often overlapped with suggestions for making students more aware of the TnT research.

*“Try and get out there a bit more, like I would hold like a presentation maybe, like sometimes people come in for, like, on our tutorial days, and they do a whole presentation on something, so maybe you could do that.”* Interviewee 428 (male, age group 16–18 years, TnT attender).

#### Publicity and reminder practicalities

Students and college staff were divided as to the best way to publicise and remind students about on-site STI testing. Some participants suggested posters and leaflets while others firmly advised against anything on paper, arguing electronic methods such as texts, emails and the college intranet were best. Conversely, some argued that many students never looked at these forms of communication and tutor general announcements were likely to be more effective.

#### The use of incentives

Students, college staff and researchers saw incentives, not necessarily money, as likely to increase uptake of STI testing. One female student offered examples of incentives not associated with sexual activity e.g. nail polish, cakes. While another female student was adamant that “*If you were to give out condoms more boys would come*”. Interviewee 069 (female, age group 16–18 years, TnT non-attender).

## Discussion

The offer of STI testing in a technical college setting was viewed positively by students. However, feelings of embarrassment and the potential of being stigmatised were deterrents. The negative influence of perceived social stigma on STI testing in college students has been reported from the US [12]. While the non-medical setting was mainly viewed as a mitigating factor against stigma, some students considered the act of volunteering to be tested exposed them to discrediting judgements by their peers. A comparative study of three types of non-medical settings in Scotland found the uptake of STI testing was lower in college than workplace or health and fitness venues [27].

The incentive of a financial gift was viewed as a facilitator to the uptake of testing. An Australian pilot study of chlamydia screening in two Universities and a technical institute reported increased uptake of 42% with a financial incentive compared to 24% uptake in a phase without a financial incentive [28]. Some female students considered an incentive that was not associated with sexual activity was important because it could not lead to discrediting judgements by peers. The positive impact of financial incentives on uptake of human immunodeficiency virus/STI testing in non-medical settings has been noted before [29]. However the role and type of incentives attractive to different sub-groups of adolescent students in technical college settings requires further investigation.

Students and researchers reported the strong influence peers had to take up, or not, STI testing. The influence of peers on adolescents’ decision making, both positive and negative, has been well documented [30]. Further work is required on how to capitalise on the positive impact of peers to increase STI testing rates.

Low levels of knowledge about STIs were perceived to have a negative effect both on the uptake of the TnT testing, and on students’ views of themselves as candidates for testing i.e. at risk [14]. Lack of STI knowledge was considered to contribute to the low priority given to STI testing by students. As in studies from Germany [31], Ireland [32], USA [33] and Australia [34] participants said sex education in school was patchy and primarily related to avoiding unwanted pregnancy rather than STIs and their possible long-term consequences. Students considered access to knowledge about STIs was important, a finding also reported in a study of young peoples’ views of new types of STI care pathways in England [35]. Given the influence of peers, peer–led education could be one option. However, there is evidence that while peer-to-peer sexual health education is effective for knowledge and attitudes, there is less evidence it impacts on actual behaviour [36]. Further research is required to investigate different mechanisms of peer influence on behaviour change outcomes related to STI testing.

There were suggestions that some teenagers considered themselves invulnerable to STIs despite engaging in risky sexual behaviours. This requires further investigation but could be explained by theories of perceived low levels of risks by adolescents as a population [37]. Some psychologists suggest that experience in the absence of negative consequences may increase feelings of invulnerability in adolescence [38]. Again, there is a need to explore the extent to which increased knowledge and peer influence might mitigate against this perception.

Finally, the study provided, in essence, a pop-up clinic for STI testing and treatment which was intermittent in nature (and often logistically challenging). Consequently, TnT was viewed as not necessarily responsive to the immediacy of student decision making and as a research study it was not intended to. However, health services in non-medical settings like technical colleges are often intermittent and the challenge is therefore to ensure safety netting for teenagers, for example, ensuring information is readily available about other local STI testing services.

### Strengths and weaknesses

This is the first qualitative research which explores the views of on-site, technical college STI testing of both males and females in an ethnically diverse group of students. Over 80% of students interviewed were aged 16–18, a group who do not often take part in sexual health studies [2]. Although most information came from participants, findings were backed by interviews with college staff and researchers. Finally, we highlight pragmatic problems of providing STI screening in a non-medical setting.

The main limitations relate to the nature of small qualitative investigations. The voluntary nature of participating meant that the voice of some sub-groups might by missing (for example bisexual young women and men), although we aimed for diversity in the purposive sample and interviewed until the point of data saturation. We have tried to ensure rigour and credibility in detailing the methods, analytic process and providing multiple perspectives. Where the views of the different participant groups have converged or supported each other we have noted this. However, we have also prioritised the students’ voice as the target group for the intervention, in keeping with our interpretive approach in which there are multiple social constructions of reality by different actors and groups [39]. Young male participants may have been inhibited by a female interviewer but techniques such as initial icebreaker questions within the interviews tried to reduce this as far as possible. Finally, findings may not be generalisable to sexually active young people from different backgrounds such as those in more traditional academic education settings, more affluent or less ethnically diverse. This requires further study.

## Conclusions

Among students with high rates of STIs who may not otherwise access testing, this study shows that TnT in college was popular with the few who attended. However, findings also suggest that future studies in similar high risk, hard to reach groups should consider linking testing with: education about STIs, offering incentives and the engagement of peer influencers. Logistical issues of STI screening in non-medical settings also need consideration. Finally, we suggest that theoretical constructs related to candidacy, peer influence and perceptions of risk in adolescence are used to frame educational interventions to accompany further research into STI screening in these populations.

## Supplementary information

**Additional file 1.** Topic Guide. Detail of questions in the interview topic guide.

**Additional file 2.** Checklist for standards for reporting qualitative research. Providing the manuscript page numbers against the standards item checklist.

## Data Availability

De-identified data sets are available from Professor Oakeshott, email oakeshot@sgul.ac.uk, subject to appropriate approvals.

## Abbreviations

CT: *Chlamydia trachomatis*
GC: *Neisseria gonorrhoeae*
NATSAL-3: National Surveys of Sexual Attitudes and Lifestyles
STI: Sexually transmitted infection
TnT: “Test n Treat” trial
UK: United Kingdom
