# Supplementary material for: Understanding the acceptability, barriers and facilitators for chlamydia and gonorrhoea screening in technical colleges: qualitative process evaluation of the “Test n Treat” trial
Source: BMC Public Health. 2020 Aug 8;20:1212. doi: 10.1186/s12889-020-09285-1 (PMC7414554; doi:10.1186/s12889-020-09285-1)
Supplement: Supplementary file 1 — Additional file 1. Topic Guide. Detail of questions in the interview topic guide. [file 12889_2020_9285_MOESM1_ESM.pdf]

Additional file 1 **Topic guide**

Guide for interviewer before recorder is switched on:

- Check the student is OK with the interview being recorded. Stress confidentiality and anonymity e.g. The interview will be typed up , but your name and college will be taken out so that your interview is completely confidential. *If participant is happy to continue, switch on recorder.* If not take notes.
- State participant ID number
- Confirm consent for recording interview

-Brief overview of the aims of the interview: to find what you think of what happened in the study and what you think other students like you would think of it.

- All feedback, good and bad is helpful, so please feel free to be as open as possible about your opinions.

Begin interview:

- *Ice breaker questions :*
  - Could you tell me first something about your studies at the college? *E.g. what course?*
  - Are you aware of any health services provide in the college? *Probe: Do you think students like you use them?*
  - Are you aware of any sexual health services at the college?
  - *Probe - Do other people your age use these services/do you think other people your age would use these services if they were available?*
- Thank you for taking part in the Test and Treat , can you tell me a bit about why did you decide to take part in Test and Treat when we first came to your college in October?
- *Do you think other students took part for the same reasons? or for other reasons ?*
- Can you tell me a bit about the reasons why you came to get tested when we visited in [add dates] ? *If did not come in [date], could you tell me a bit about why you decided not to come in[date]?*
- Could you tell me what did you think about the service we provided?(*prompts – was it as you expected ? did you understand what was happening ? could we have done anything differently ?*)
- What do you think would have encourage more people taking part in the study to come and get tested when we were at college?
  - *Do you think there is anything (else) that might be putting other people (your age/at your college) off coming and getting tested?*
  - *Is there anything that we could change?*
- What do you think people your age/at college think about going to the sexual health clinic/ clinic?
- Do you think you think that other students your age know about sexually transmitted infections like chlamydia and gonorrhoea?
- Can you tell me what you know about chlamydia and gonorrhoea?
- Overall what do you think is good about the Test and Treat service that has been offered in this study ?

- Anything not so good that could be improved?
- If we did this Test and Treat service in another college what could we do differently that would encourage students to use it?
- Any other comments?

Thank you
