# Supplementary material for: Understanding the acceptability, barriers and facilitators for chlamydia and gonorrhoea screening in technical colleges: qualitative process evaluation of the “Test n Treat” trial
Source: BMC Public Health. 2020 Aug 8;20:1212. doi: 10.1186/s12889-020-09285-1 (PMC7414554; doi:10.1186/s12889-020-09285-1)
Supplement: Supplementary file 2 — Additional file 2. Checklist for standards for reporting qualitative research. Providing the manuscript page numbers against the standards item checklist. [file 12889_2020_9285_MOESM2_ESM.docx]

**Additional file 2. Checklist for standards for reporting qualitative research** ^1^

| Item |  | Description | Addressed on page number in manuscript |
| --- | --- | --- | --- |
| 1 | TITLE | Concise description of the nature and topic of the study. Identifying the study as qualitative | 1 |
| 2 | ABSTRACT | Summary of key elements of the study using the abstract format of the intended publication; | 3 |
| 3 | Problem Formulation | Description and significance of the problem/phenomenon studied; review of relevant theory and empirical work; problem statement. | 5 |
| 4 | Purpose or research question | Purpose of the study and specific objectives or questions. | 5 |
| 5 | Qualitative approach and research paradigm | Qualitative approach; identifying the research paradigm (e.g., post-positivist, constructivist/interpretivist) | 6 |
| 6 | Researcher characteristics and reflexivity | Researchers’ characteristics that may influence the research. | 7 and 18 |
| 7 | Context: | Setting/site and salient contextual factors; rationale. | 5 and 6 |
| 8 | Sampling strategy | How and why research participants, documents, or events were selected; criteria for deciding when no further sampling was necessary (e.g., sampling saturation); rationale. | 6 |
| 9 | Ethical issues pertaining to human subjects | Documentation of approval by an appropriate ethics review board and participant consent, or explanation for lack thereof; other confidentiality and data security issues. | 20 |
| 10 | Data collection methods: | Types of data collected; details of data collection. | 6 |
| 11 | Data collection instruments and technologies | Description of instruments (e.g., interview guides, questionnaires) and devices (e.g., audio recorders) | 6 |
| 12 | Units of study | Number and relevant characteristics of participants, documents, or events included in the study; level of participation. | 8 and Table 1 |
| 12 | Data processing | Methods for processing data prior to and during analysis, including transcription. | 6 |
| 14 | Data analysis: | Process by which inferences, themes, etc. were identified and developed, including the researchers involved in data analysis | 7-8 |
| 15 | Techniques to enhance trustworthiness | Techniques to enhance trustworthiness and credibility of data analysis,(e.g., member checking, triangulation, audit trail); rationale. | 7-8 |
| 16 | Synthesis and interpretation | Main findings (e.g., interpretations, inferences, and themes. | 8 |
| 17 | Links to empirical data | Evidence (e.g., quotes, field notes, text excerpts, photographs) to substantiate analytic findings. | 8-16 |
| 18 | Integration with prior work, implications, transferability, and contribution(s) to the field | Short summary of main findings, explanation of how findings and conclusions connect to, support, elaborate on, or challenge conclusions of earlier scholarship; discussion of scope of application/generalizability; identification of unique contribution(s) to scholarship in a discipline or field. | 16-17 |
| 19 | Limitations | Trustworthiness and limitations of findings | 18 |
| 20 | Conflicts of interest | Potential sources of influence or perceived influence on. | 19 |
| 21 | Funding | Sources of funding and other support; role of funders in data collection, interpretation, and reporting. | 19 |

^1^ O'Brien BC, Harris IB, Beckman TJ, Reed DA, Cook DA. Standards for reporting qualitative research: a synthesis of recommendations. Acad Med. 2014;89(9):1245-1251.
